# Comparative IP-MS Reveals HSPA5 and HSPA8 Interacting with Hemagglutinin Protein to Promote the Replication of Influenza A Virus

**DOI:** 10.3390/pathogens14060535

**Published:** 2025-05-27

**Authors:** Xingwei Feng, Mengfei Ning, Bin Chen, Xuan Li, Honglei Sun, Juan Pu, Jinhua Liu, Na Wang, Yinhua Huang

**Affiliations:** 1 State Key Laboratory of Animal Biotech Breeding, College of Biological Sciences, China Agricultural University, No. 2 Yuan Ming Yuan West Road, Hai Dian District, Beijing 100193, China; xingweifeng128@126.com (X.F.); 18811070258@163.com (M.N.); sz20233020336@cau.edu.cn (B.C.); hacet724@163.com (X.L.); 2 Frontiers Science Center for Molecular Design Breeding (MOE), China Agricultural University, Beijing 100193, China; 3 Key Laboratory for Prevention and Control of Avian Influenza and Other Major Poultry Diseases, Ministry of Agriculture and Rural Affairs, College of Veterinary Medicine, China Agricultural University, Beijing 100193, China; shlei668@163.com (H.S.); pujuan@cau.edu.cn (J.P.); ljh@cau.edu.cn (J.L.)

**Keywords:** influenza A virus (IAV), Hemagglutinin (HA), immunoprecipitation–mass spectrometry (IP-MS), HSPA5 and HSPA8

## Abstract

The influenza A viruses (IAV) are the principal pathogens for annual (seasonal) influenza, which cause world-wide outbreaks in poultry and pose a persistent threat to public health. The Hemagglutinin protein (HA) of IAV promotes virus infection by binding the host membrane receptor and mediating virus–host membrane fusion. Immunoprecipitation–mass spectrometry (IP-MS) provides global insights into IAV HA–host protein interactions. However, various experimental conditions might affect the identification of interactions. Here, we performed a serial IP-MS to compare interactors of IAV HA in accidental host human, chicken and reservoir host duck cells. We found that the positive ratio of interactors identified by the IP-MS was improved when the transfected HA plasmid had a similar expression level to HA proteins found in IAV virus infection. Comparing interactors in human, chicken and duck cells, we found that HA–interacting host factors might play a role in the susceptibility of accidental hosts (human and chicken) to IAV infection compared to reservoir hosts (duck). We then focused on the function of two heat shock proteins (HSPA5 and HSPA8), which interacted with IAV HA proteins in all three species (human, chicken and duck). We found that both HSPA5 and HSPA8 promoted the IAV replication by enhancing the viral attachment and internalization. These findings extend our knowledge about the mechanisms of IAV entry to host cells and provide target genes to create chickens resistant to avian influenza.

## 1. Introduction

Influenza A virus (IAV) is the principal pathogen for annual (seasonal) influenza, causing disastrous disease in poultry and posing a substantial threat to human health [1]. IAV is an enveloped virus of the *Orthomyxoviridae* family with eight single-stranded, negative-sense RNAs genomes encoding 10–11 essential proteins and a few accessory proteins [2,3]. Hemagglutinin protein (HA) is the most abundant glycoprotein on the influenza virus membrane. It initiates IAV infection through binding to the sialic acid (SA) receptor on host cell surface glycolipids or glycoproteins, and subsequently promotes the virus’ internalization through endocytosis, including clathrin-dependent and clathrin-independent modes, or micropinocytosis [4]. HA also plays a critical role in mediating the fusion between the viral envelope and endosomal membrane, thus accelerating the entering of viral ribonucleoprotein into the host cell cytoplasm [5]. Previous studies have indicated that several host factors, including epidermal growth factor receptor (EGFR) [6], free fatty acid receptor 2 (FFAR2) [7], voltage-dependent Ca^2+^ channel (Cav1.2) [8] and mGluR2 [9], are involved in the attachment or internalization by interacting with HA during IAV entry. Therefore, exploring the mechanism of interaction between HA and other host factors can further reveal the biological details underlying IAV’s entry process and pathogenic mechanisms of IAV.

Several proteome-wide screens have been performed to identify host factors that interact with IAV HA by using affinity purification [10,11], immunoprecipitation followed by mass spectrometry (IP-MS) [12] or yeast two-hybrid techniques [13]. Especially, IP-MS is widely used due to its accuracy, sensitivity and ease of operation. However, when screening for viral interactions with host factors, the issues it reveals indicate that there is still potential for optimization. Due to the difficulties in obtaining specific antibodies against viral proteins and their low affinity characteristics, researchers usually resort to using overexpression plasmids to enrich virus–host protein interaction complexes via tagged antibodies as a prerequisite for screening interacting factors. However, the use of plasmids often leads to excessively high levels of protein expression, which can disrupt the pathological environment and result in false positives during the screening process. Here, we conducted a comparative screen based on the IP-MS to identify the host factors interacting with the HA protein of IAV and plasmids. This screen allows us to investigate the effects of various IP-MS methods on the detection of host factors interacting with IAV HA. Comparing IAV HA–different host protein interactions, we found that the IAV HA-interacting host factors might contribute to the susceptibility of accidental hosts (human and chicken) to the influenza virus. Moreover, we demonstrated that HSPA5 and HSPA8 interact with IAV HA and promote IAV replication by enhancing the viral attachment and internalization processes in chicken fibroblast cells, providing candidate target genes for the molecular breeding of avian influenza-resistant chickens.

## 2. Materials and Methods

### 2.1. Cells and Virus

Duck embryo fibroblast primary cells (DEF), Chicken embryo fibroblast primary cells (CEF), Chicken embryonic fibroblasts cells (DF1), Human embryonic kidney cells (HEK293T) and Madin–Darby canine kidney cells (MDCK) were cultured in Dulbecco’s modified Eagle’s medium (DMEM; Gibco, Carlsbad, CA, USA) supplemented with 10% fetal bovine serum (FBS; Gibco, Carlsbad, CA, USA) at 37 °C in a 5% CO_2_ humidified incubator. All media contained 100 U/mL penicillin and 100 µg/mL streptomycin. DEF and CEF were isolated and cultured from 9-day-old SPF chicken (Boehringer Ingelheim, Beijing, China) and duck (Seed Industry Group, Jinan, China) embryos. H1N1 virus (A/Puerto Rico/8/34, PR8), H5N1 virus (A chicken/huabei/0513/2007, CK/0513), H3N8 virus (A/chicken/Anhui/FE12/2022, H3N8) and H9N2 virus (A/chicken/guangdong/F93/2022, H9N2) were propagated in 10-day-old chicken embryos.

### 2.2. Plasmids

The open reading frames (ORFs) of the *HA* genes of PR8 H1N1 and CK/0513 H5N1 virus were cloned into *psp72* expression vectors containing a Flag tag at the C terminus. Chicken *HSPA5* and *HSPA8* CDs regions were amplified from total RNA of chicken lungs infected with the CK/0513 H5N1 virus and cloned into *pPB-neo* expression plasmid with a Flag tag at the C-terminus. The CDs region of 30 chosen IAV HA interacting proteins in HEK293T cells were cloned and inserted into the *psp72* expression plasmid bearing a Flag tag at the C-terminus. All plasmid constructs were verified by sequencing. The primer sequences used for the generation of all constructs are shown in Table S2. Chicken *HSPA5* cDNA serial number—XM_0406489802, chicken HSPA8 cDNA serial number—NM_205003.3. The sequences of the H5N1 *HA* and H1N1 *HA* cDNA are shown in Supplementary Table S1.

### 2.3. Antibodies

The commercially obtained primary antibodies used in this study were as follows: mouse anti-Flag monoclonal antibody (mAb) (MA1-91878, Thermo, Waltham, MA, USA), rabbit anti-GAPDH mAb (MA1-16757, Thermo, Waltham, MA, USA), mouse anti-IgG mAb (A7028, Beyotime, Shanghai, China), rabbit anti-IgG mAb (A7016, Beyotime, Shanghai, China), rabbit anti-H5N1 HA mAb (40158-T62, SinoBiological, Beijing, China), rabbit anti-H1N1 HA mAb (11648-T62, Sino Biological, Beijing, China) and mouse anti-NP mAb (ab20343, Abcam, Cambridge, UK). The secondary antibodies used for Western Blotting and Immunofuorescence assay included anti-mouse IgG (H + L), human serum adsorbed and FITC-labeled (5230-0427, LGC, Teddington, UK), goat anti-rabbit IgG H&L (HRP) (ab6721, Abcam, Cambridge, UK), and goat anti-mouse IgG H&L (HRP) (ab205719, Abcam, UK).

### 2.4. Protein Immunoprecipitation

For IP-MS, HEK293T cells were transfected with a plasmid expressing the PR8 HA protein or infected with PR8 virus at 1 MOI. DEF and CEF cells were transfected with a plasmid expressing CK/0513 HA protein or infected with CK/0513 virus at 0.1 MOI. Cell transfection used the jetPRIME transfection reagent (Polyplus Strasbourg, France). For Co-IP, the plasmids expressing HA and HSPA5-Flag, HSPA8-Flag or 30 host protein fusing Flag tag were contransfected into DF1 or HEK293T cells. After treatment for 48 h, the above cells were lysed in IP lysis buffer (Beyotime, Shanghai, China) containing a final concentration of 1 mM PMSF (Beyotime, Shanghai, China) for 40 min. The lysate was cleared using protein A+G agarose (Beyotime, Shanghai, China) and specific IgG for 2 h at 4 °C and then incubated with specific antibody (anti-H1N1-HA, anti-H5N1-HA, anti-IgG) and protein A+G agarose overnight at 4 °C. After that, the immunoprecipitated proteins were analyzed using SDS-PAGE following silver staining and a mass spectrometry (MS) analysis at the Institute of Microbiology (Chinese Academy of Sciences) to identify coimmunoprecipitated host proteins, or Western Blotting to identify the interaction between proteins.

### 2.5. Western Blotting

The coimmunoprecipitated protein samples were separated by SDS-PAGE and transferred onto the polyvinylidene difluoride membranes. The membranes were blocked with 5% nonfat milk in PBST buffer for 2 h, incubated with the specific primary antibodies overnight at 4 °C, washed, and incubated with the appropriate horseradish-conjugated secondary antibody for 1 h at room temperature. The bolts were visualized using the enhanced chemiluminescene method, followed by exposure in a dark room.

### 2.6. ShRNA Transfection

DF1 cells were grown in a 12-well plate with DMEM containing 10% FBS at a density reaching 70% confluency within 24 h, and the cells were either transfected with 1 μg specific shRNA (*HSPA8* shRNA, *HSPA5* shRNA) or scramble shRNA using 3 µL of Lipofectamine 2000 (Thermo Fisher, Carlsbad, CA, USA). Transfected cells were incubated under standard conditions at 37 °C with 5% CO_2_ for 24 h. The shRNAs used in this study are listed in Table S2.

### 2.7. Cell Viability Assay

Cell viability was measured using a Cell Counting Kit-8 (CK04, DOJINDO, Kumamoto, Japan) according to the manufacturer’s instructions. Briefly, DF1 cells were seed on 96-well plates and transfected with shRNA targeting *HSPA5*, *HSPA8* or scrambled shRNA for 12, 24, 36 and 48 h. After that, 10 µL of CCK8 reagent was added and cultured for 4 h at 37 °C, and then the absorbance of each sample was measured at a wavelength of 450 nm with the microplate reader.

### 2.8. Virus Infection

DF1 were transfected with shRNA targeting *HSPA5*, *HSPA8* or scrambled shRNA, and at 24 h post-transfection, the cells were infected with H5N1, H3N8 or H9N2 virus at an MOI of 0.1. At 12, 24, 36 and 48 h post-infection (p.i.), the supernatants were collected and titrated for infectious viruses by tissue culture infective dose (TCID_50_) in MDCK. Three independent experiments were performed.

### 2.9. Virus Attachment and Internalization Assay

To examine the effects of HSPA5 or HSPA8 on IAV attachment, DF1 cells in 6-well plates were treated with shRNA targeting *HSPA8*, *HSPA5* or scrambled shRNA for 24 h and then infected with CK/0513 H5N1 virus at an MOI of 1 at 4 °C for 2 h. The infected cells were washed five times with ice-cold PBS (7.2) and collected for RT-qPCR.

To examine the effect of chicken HSPA5 or HSPA8 on IAV internalization, DF1 cells in a 6-well plate were treated with shRNA targeting *HSPA8*, *HSPA5* or scrambled shRNA for 24 h and then infected with CK/0513 H5N1 virus at an MOI of 1 at 4 °C for 2 h, followed by a culture temperature shift to 37 °C for 30 min to allow for internalization. The infected cells were washed five times with ice-cold PBS (2.5) to remove the attached but not-yet-internalized virions and collected for RT-qPCR [14].

### 2.10. Real Time qPCR (RT-qPCR)

Total RNA was isolated from the cells using Trizol reagent. cDNA was synthesized with the HighCapacity cDNA Reverse Transcription Kit (Invitrogen, Carlsbad, CA, USA). All RT-qPCR were performed using the LightCycler 480 SYBR Green I Master Mix on a detection system (Roche, Basel, Switzerland) with primers in Table S2. For analysis of the *NP* copy number of the attached or internalized CK/0513 virus, the standard curves generated according to the threshold cycle (Ct) of known concentrations and the virus copy number of each sample were converted using the standard curve. For estimation of the knockdown efficiency, the *HSPA5*- or *HSPA8*-specific Ct values were normalized to that of the *GAPDH*. Gene differential expression between samples was calculated using the 2^−ΔΔCT^ method [14].

### 2.11. Immunofuorescence Assay

DF1 cells were seeded in glass cover slips at a density that allowed 80% confluency to be reached within 24 h, at which the cells were transfected with the shRNA targeting *HSPA5*, *HSPA8* or scrambled shRNA by using the Lipofectamine 2000 Reagents. After 24 h, cells were infected with CK/0513 H5N1 virus at an MOI of 1. At 4, 6, and 8 h p.i., the cells were washed twice with PBS and fixed with 1% paraformaldehyde in 1× PBS for 1 h. After removal of the reagent, the cells were permeabilized with 0.1% Triton X-100 in PBS at 37 °C for 15 min (membrane surface proteins are detected without this permeability treatment), and then blocked with 10% BSA in PBS for 1 h followed by incubation with two different primary antibodies (mouse anti-NP mAb, 1:400) at 4 °C overnight. The cells were washed three times with PBS and incubated with the secondary antibodies for 1 h. After being washed with PBS four times, the cells were incubated with DAPI (4′,6-diamidino-2-phenylindole, Thermo Fisher Scientific, Waltham, MA, USA) for 15 min to stain the nuclei (membrane surface proteins are detected without this nuclear staining). Images were acquired using the Nis A1 confocal system (Nikon Corporation, Tokyo, Japan).

### 2.12. Mass Spectrometry Analysis

DataAnalysis 4.0 was used to interpret MS spectra and generate peak tables. A MASCOT search was performed on the latest Swiss-Prot protein identification database using MASCOT 2.2.06 software. The search parameters were set as follows: enzyme selected as trypsin with one maximum missing cleavage site, modification of carbamidomethyl (C) and variable modification of oxidation (M). The species were classified as human, chicken and duck. The peptide mass tolerance was 10 ppm and fragment mass tolerance was 0.6 Da. Positive protein identifications were based on a significant MOWSE score. MOWSE was used to score positive proteins and a fault tolerant search was performed to detect non-specific cleavage. The identified proteins were examined and filtered manually, and the protein obtained from the IgG group was used as a control to remove redundant proteins and obtain specific interacting proteins.

### 2.13. Gene Ontology

Gene Ontology (GO) enrichment was performed using Database for Annotation, Visualization and Integrated Discovery (DAVID) (https://david.ncifcrf.gov/ (accessed on 24 June 2024)), and we limited the species to human, chickens and ducks. GO enrichment analysis was performed on potential genes involved in viral life cycle and immunity, and important signaling pathways for HA to interact with host proteins to activate cellular immunity or assist viral immune escape were screened. The results were screened for *p* values of <0.05.

### 2.14. Statistical Analysis

In this study, statistical analysis and presentation graphics were produced with GraphPad Prism 5.01 software (GraphPad). The results are shown as mean ± SEM and represent data from at least three independent experiments. Two-tailed Student’s *t*-test was used for statistical significance. The *p* values < 0.05 are deemed to denote statistically significant difference, and the significance levels were as follow: * *p* < 0.05; ** *p* < 0.01; *** *p* < 0.001.

## 3. Results

### 3.1. Identification of the Host Factors That Interacted with HA Protein of IAV and Plasmids

**Figure 1:**
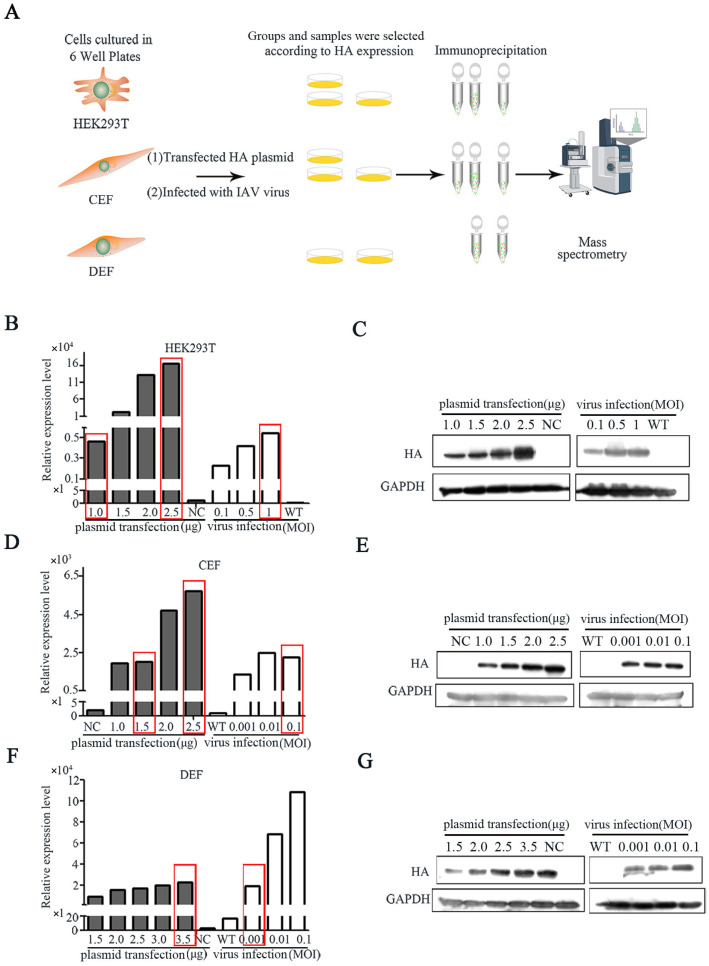
Screening strategy for comparing host factors interacting with HA from IAV and plasmids. (**A**) IP-MS experimental workflow for identifying interactions between HA and host proteins from human HEK293T, chicken CEF and duck DEF cells. HEK293T cells were transfected with H1N1 *HA* plasmid or infected with H1N1; CEF and DEF cells were transfected with H5N1 *HA* plasmid or infected with H5N1. (**B**,**D**,**F**) HA expression was detected by RT-qPCR and Western Blotting (**C**,**E**,**G**) in HEK293T, CEF and DEF cells were transfected with plasmid or infected with IAV. The red boxes are the selected samples (plasmid, high and virus) from each species.

**Figure 2:**
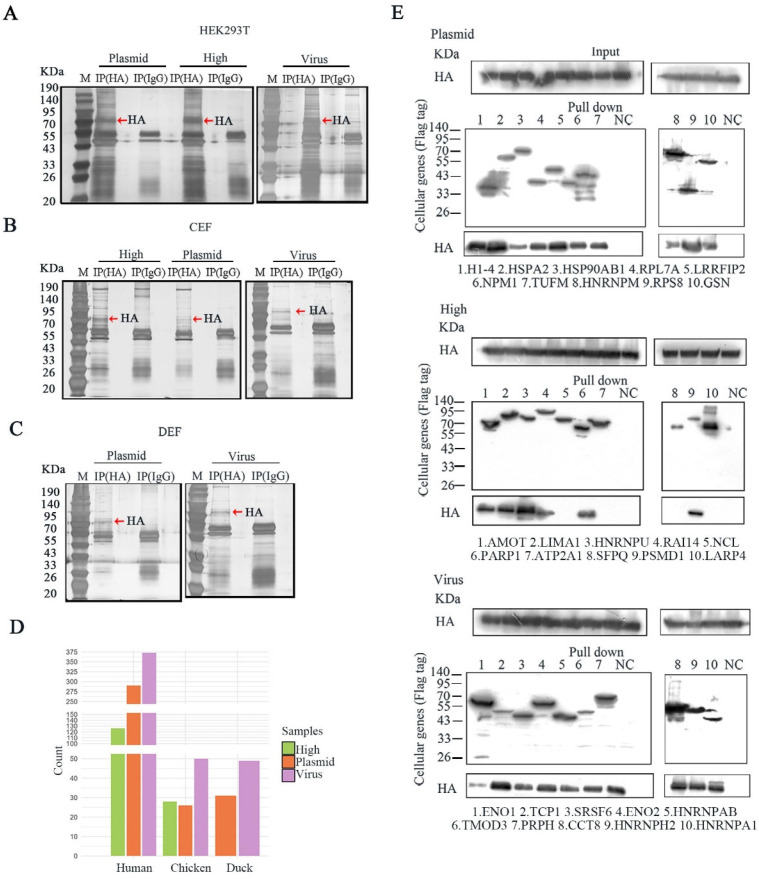
Identification of the host factors interacting with HA protein of IAV and plasmids. (**A**–**C**) HA protein enrichment was detected by silver staining assay in the selected samples (high, plasmid and virus) from HEK293T, CEF and DEF cells, with IgG antibodies as the control. The red arrow refers to the enriched HA protein. (**D**) The number of HA interaction factors screened in different categories (high, plasmid and virus) of human, chicken and duck species was counted in the bar chart. (**E**) Interactions between HA and indicated cellular binders were examined by immunoprecipitation (IP)-Western Blotting. HA and one of the Flag-tagged cellular proteins were co-expressed in HEK293T cells. Cells were lysed 48 h post transfection. Cellular proteins were pulled down with Flag beads and detected for co-eluted HA using an antibody against HA.

We developed a screening method to systematically compare the HA–host protein interactions in IAV and plasmids (Figure 1A). In the plasmid transfection group, human HEK293T, chicken CEF and duck DEF cells were transfected with the *HA* plasmid at different concentrations of 1.0, 1.5, 2.0, 2.5, 3.0 and 3.5 μg for 48 h, with an empty NC plasmid as negative control. In the virus infection group, these above cells were infected with influenza virus at different multiplicities of infection (MOI) of 0.001, 0.01, 0.1, 0.5 and 1 for 24 h, with uninfected wild-type (WT) cells as a negative control. HA expression in mRNA (Figure 1B,D,F) and protein levels (Figure 1C,E,G) showed dose-dependent increases with the increase in the amount of *HA* plasmid transfection or virus infection. The samples with similar HA expressions derived from plasmid transfection (Plasmid) and virus infection (Virus) were selected for the immunoprecipitation analysis: HEK293T cells were transfected with 1.0 μg of plasmid and infected at an MOI of 1; CEF cells were transfected with 1.5 μg of plasmid and infected at an MOI of 0.1; DEF cells were transfected with 3.5 μg of plasmid and infected at an MOI of 0.001 (Figure 2A–C). Moreover, HEK293T cells and CEF cells transfected with 2.5 μg resulting in high HA expression (High) were chosen to analyze the effects of different expression levels of HA on the coprecipitated host proteins (Figure 2A,B); 2.5 μg is the recommended normal transfection dose according to the instructions.

**Figure 3:**
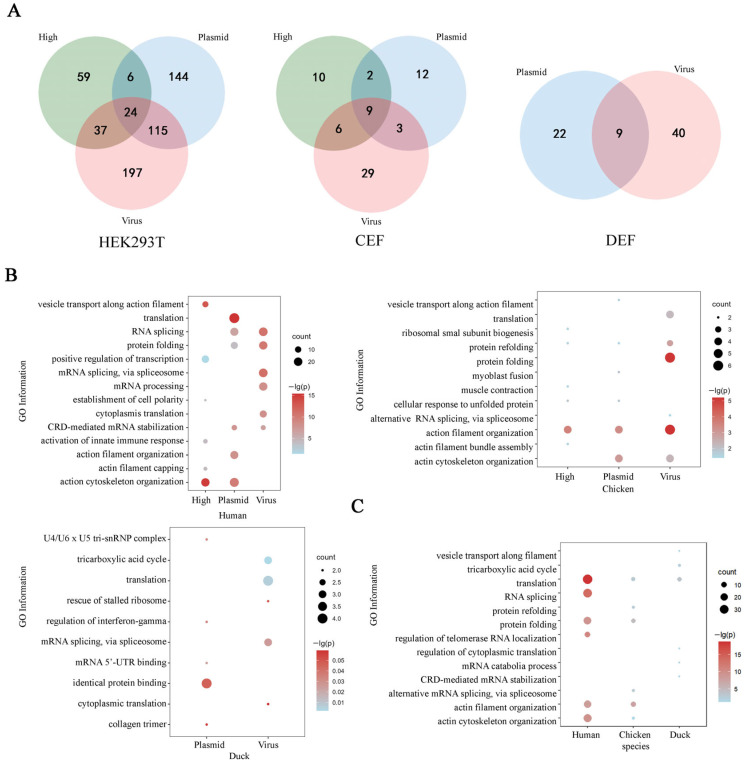
Functional analysis of HA-interacting host proteins. (**A**) Venn diagram showing the overlap of HA-interacting host proteins identified in samples (high, plasmid and virus) of human, chicken and duck species. (**B**) GO enrichment analysis of genes that were identified to be interacting with HA in samples (high, plasmid and virus) of human, chicken and duck species. The database for Annotation, Visualization and Integrated Discovery (DAVID) was applied for this analysis. (**C**) GO enrichment analysis of HA-interacting host proteins in species of human, chicken and duck. The database for Annotation, Visualization and Integrated Discovery (DAVID) was applied for this analysis.

We next analyzed the mass spectrometry (MS) data from the immunoprecipitated samples to identify 582, 71 and 71 non-redundant host proteins interacting with IAV HA in HEK293T, CEF and DEF cells, respectively (Table S1). In the plasmid transfection group, the HA-host interactions were irregular when the expression of HA was increased, because the increased amount of transfected HA disrupts the normal pathological environment, resulting in more or fewer interacting proteins being screened (Figure 2D). When the expression of HA was similar, the virus-infected sample had a significantly larger number of interacting host proteins than the plasmid-transfected sample across different cell types-373 versus 289 in HEK293T, 47 versus 26 in CEF and 49 versus 31 in DEF cells (Figure 2D). However, the overlaps of HA-host interaction between the virus-infected group and the plasmid-transfected group were few (Figure 3A).

We further evaluated the positive interaction ratio of identified HA-host protein interactions among different samples. Ten proteins interacting with HA from each HEK293T cell sample (high, plasmid and virus) were chosen according to their IP-MS score, except overlaps. The plasmids expressing host protein were cotransfected with *HA* and interactions were tested by co-immunoprecipitation, using the cotransfection of HA and empty NC plasmid as a negative control. This test indicated that all ten host proteins were interacted with HA in the plasmid and virus samples, whereas interactions were only observed in six host proteins from the high sample. Therefore, the positive rates of the three samples were 100%, 100% and 60% (high, plasmid and virus, respectively). This indicates that the positive rate of interacting protein in the plasmid sample was higher than that in the high sample, and there were more false positives in the high sample (Figure 2E).

In summary, for IP-MS, the expressional level of baited HA influences the number and false positive ratio of coprecipitated host proteins, and there was a limited overlap between the plasmid-transfected and virus-infected group, with similar expressions of HA.

### 3.2. Functional Analysis of HA-Interacting Host Proteins

We investigated the differences in the biological functions of host factors interacting with the HA protein of IAV and plasmids. Gene Ontology (GO) analysis showed that there was a limited overlap of biological functions for HA-interacting host factors identified in both groups (Figure 3B). For HEK293T and CEF cells, the host factors from the plasmid-transfected group were enriched in the compartment of the cytoskeleton, including actin cytoskeleton organization and actin filament organization/capping, but those from the virus-infected group were mainly associated with RNA and protein processes, such as mRNA splicing, protein folding and translation. For DEF cells, the host factors in the plasmid-transfected group were involved in mRNA binding and protein binding, and those from virus-infected group were related to tricarboxylic acid cycle, translation and mRNA splicing. This observation, together with the low proportion of overlap interactors in the plasmid-transfected and virus-infected groups, suggests that the experimental method of the IP-MS screen was unstable and influenced the identification of host proteins.

For the biological function of HA-interacting host proteins, human and chicken were similar and enriched in cytoskeleton (MYO1B, ACTN1, TPM1) and protein folding (HSPA5, HSPA8, HSPA9) (Figure 3C). These likely reflect the interactions between HA and cytoskeletal proteins, and might contribute to the rapid processes of viral entry, assembly and release in the accidental host of IAV. But the HA-interacting host proteins in duck were enriched in translation (EEF1A, RPS6), tricarboxylic acid cycle and mRNA splicing (EFTUD2, LSM4) (Figure 3C), indicating intracellular components interacting with HA might play a role in controlling viral replication and antiviral gene expression in the reservoir host of IAV.

### 3.3. HSPA5 or HSPA8 Interacts with HA in Chicken, Duck and Human Cells

**Figure 4:**
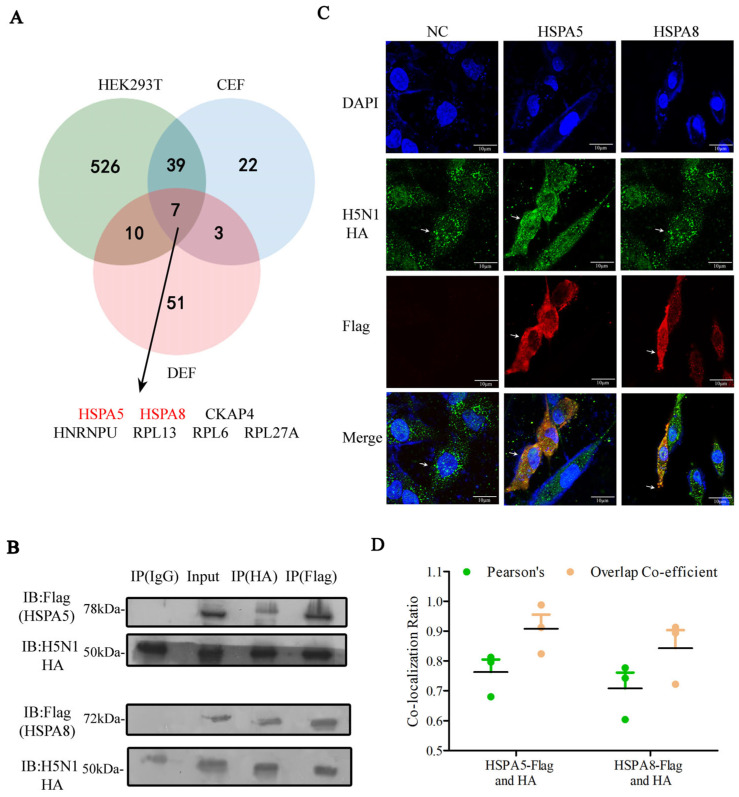
HSPA5 or HSPA8 interacts with IAV HA protein. (**A**) Venn diagram showing the overlap of HA-interacting host proteins identified in human, chicken and duck. (**B**) Interactions between HSPA5 or HSPA8 and H5N1 HA in DF1 cells were examined by immunoprecipitation at 48 h after co-transfection. (**C**) Colocalization of H5N1 HA and HSPA5 or HSPA8 in DF1 cells was monitored at 48 h after co-transfection. DAPI (blue) for nuclei visualization. Colocalization (yellow) between HSPA5 or HSPA8 (red) and HA (green) was observed under a Nikon confocal microscope. (**D**) The Pearson’s correlation and overlap coefficient are shown, deduced from three independent experiments. *n* = 3.

The HA protein of IAV is synthesized in the endoplasmic reticulum (ER), where molecular chaperone proteins are widely expressed and play a key role in protein synthesis and folding, and the quality control of processing proteins [15]. Based on our IP-MS data, only seven host proteins were commonly identified in human, chicken and duck, among which HSPA5 and HSPA8 were confirmed as high-confidence interactors of the HA protein (Figure 4A). We were thus interested in investigating the interaction between HA and HSPA5 or HSPA8. We transfected the plasmid expressing chicken Flag-tagged *HSPA5* or Flag-tagged *HSPA8* into chicken DF1 cells for 24 h and then infected them with CK/0513 H5N1 virus for 24 h, using the empty NC plasmid as the negative control. The co-immunoprecipitation (Co-IP) assay showed that HA interacted with chicken HSPA5 or HSPA8 (Figure 4B). The immunofluorescence assay also confirmed the interaction between HA and HSPA5 or HSPA8 in DF1 cells (Figure 4C,D). In addition, the localization of HSPA5 and HSPA8 in the cytoplasm is also consistent with previous reports [16,17,18].

### 3.4. HSPA5 or HSPA8 Positively Regulate the IAV Replication

**Figure 5:**
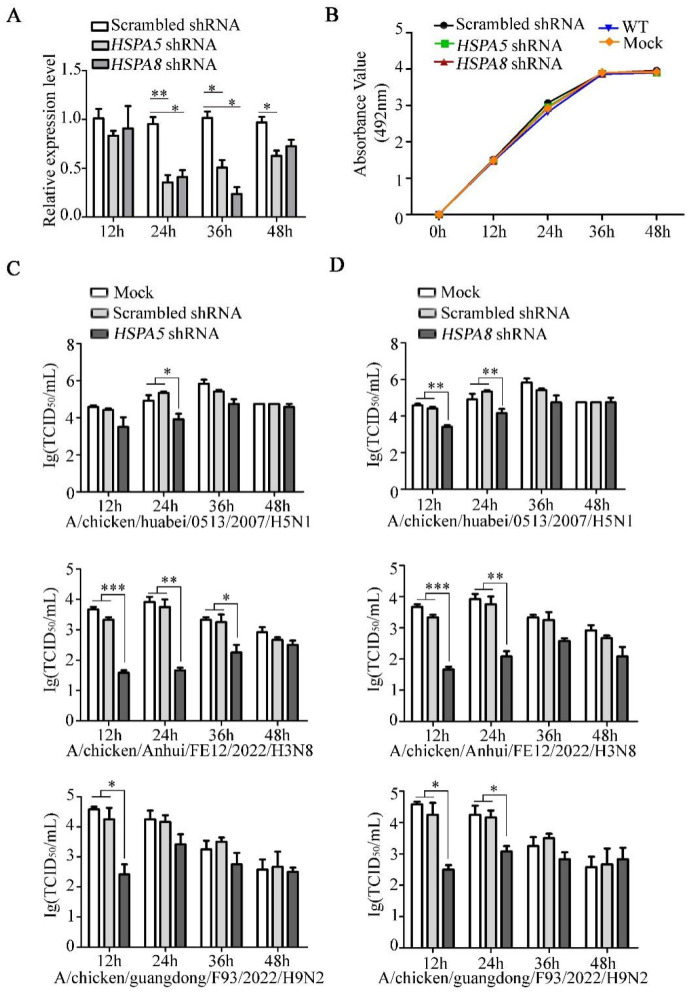
HSPA5 or HSPA8 positively regulate the IAV replication. (**A**) The knockdown of *HSPA5* or *HSPA8* in DF1 cells transfected with specific or scrambled shRNA for 12 h, 24 h, 36 h and 48 h was detected by RT-qPCR. *n* = 3, *, *p* < 0.1; **, *p* < 0.01; ***, *p* < 0.001. (**B**) Cell viability was determined using a CCK8 assay, with WT (wild-type) and Mock (Lipofectamine -only) DF1 cells as controls. *n* = 3, *, *p* < 0.1; **, *p* < 0.01. *HSPA5* shRNA- (**C**), *HSPA8* shRNA- (**D**) or scrambled shRNA-transfected DF1 cells and Mock (Lipofectamine only) DF1 cells were infected with H3N8 (MOI = 0.1), H9N2 (MOI = 0.1) or H5N1 (MOI = 0.1) virus. Virus titers at 12 h, 24 h, 36 h and 48 h were determined by means of TCID_50_ on MDCK cells. *n* = 3, *, *p* < 0.1; **, *p* < 0.01.

To investigate the function of HSPA5 and HSPA8 in IAV replication, we performed *HSPA5* or *HSPA8* gene knockdown in DF1 cells using short hairpin RNA (shRNA). Real-time PCR confirmed that the expression of *HSPA5* or *HSPA8* was efficiently reduced in specific shRNA treatment compared to the scrambled shRNA (Figure 5A). The downregulation of *HSPA5* or *HSPA8* did not affect the cell viability as measured by the Cell Counting Kit-8 (CCK8) assay (Figure 5B). We further compared the replication of high-pathogenic H5N1 IAV (CK/0513) in DF1 cells with *HSPA5*- or *HSPA8*-specific and scrambled shRNA treatment. This result showed that the knockdown of *HSPA5* or *HSPA8* significantly decreased the virus titers compared to the scrambled shRNA-treated DF1 cells. Moreover, the knockdown of *HSPA5* or *HSPA8* significantly reduced the replication of two low-pathogenic IAVs (H3N8 and H9N2) in DF1 cells (Figure 5C,D).

Collectively, HSPA5 and HSPA8 are involved in the IAV infection, and positively regulate IAV replication in DF1 cells.

### 3.5. HSPA8 or HSPA5 Is Involved in the Early Stage of IAV Replication Cycle

**Figure 6:**
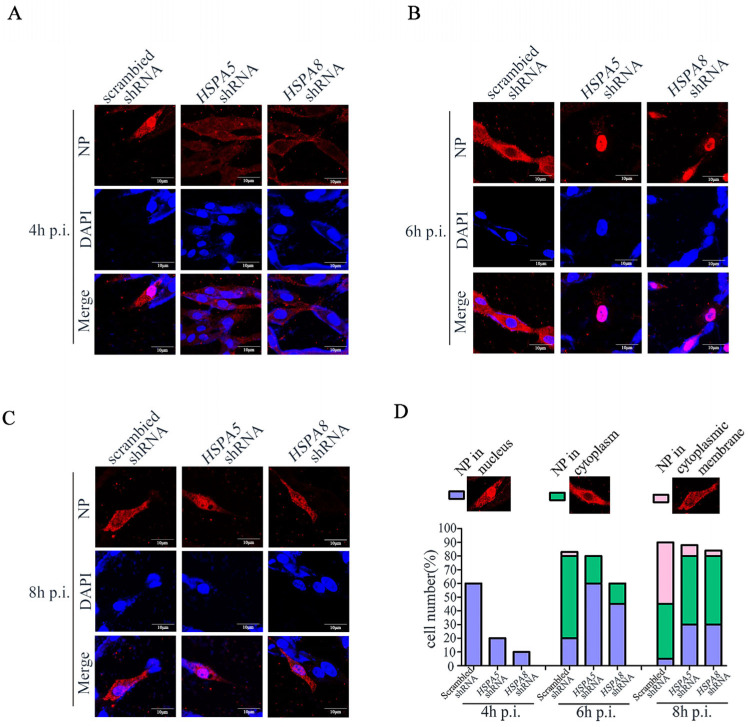
HSPA8 or HSPA5 is involved in the early stage of the IAV replication cycle. *HSPA5* shRNA-, *HSPA8* shRNA- or scrambled shRNA-transfected DF1 cells were infected with CK/0513 virus at an MOI of 1. Localization of NP in DF1 cells was monitored at 4 h (**A**), 6 h (**B**) and 8 h (**C**) by means of Immunofluorescence assay. (**D**) Quantitative analysis of NP localization in virus-infected cells; for each bar graph 100 cells were taken for analysis.

To further elucidate how HSPA5 and HSPA8 affect the IAV replication cycle, the DF1 cells transfected with *HSPA5*- or *HSPA8*-specific and scrambled shRNA were infected with CK/0513 (H5N1) virus. The cellular distribution of the viral NP protein during the virus life cycle was visualized by confocal microscopy. We found that at 4 h post-infection (p.i.), NP in the nucleus were observed in 60% of scrambled shRNA-treated cells, but only detected in 20% of *HSPA5*-specific treated cells and 10% of *HSPA8*-specific shRNA-treated cells. This observation indicates that the knockdown of *HSPA5* or *HSPA8* inhibited the early stage of the virus replication cycle (Figure 6A,D). At 6 h p.i., viral NPs were already localized in the perinuclear region and cytoplasm in 63% of scrambled shRNA-treated cells, suggesting that the new vRNPs complex was synthesized and exported to the nuclei. However, viral NPs were clearly accumulated in the nuclei of the *HSPA5*- and *HSPA8*-specific shRNA-treated cells, with only a small amount of exucleation (20% and 15%) (Figure 5B,D). At 8 h p.i., viral NPs were detected in the cytoplasmic membrane in 45% of scrambled shRNA-treated DF1 cells. In contrast, most viral NPs were still observed in the perinuclear region and cytoplasms in the *HSPA5*- and *HSPA8*-specific shRNA-treated cells, with only 8% and 4% in the cytoplasmic membrane (Figure 6C,D).

Together, these observations indicate that HSPA5 or HSPA8 was involved in the early stage of IAV, and the knockdown of *HSPA5* or *HSPA8* suppressed IAV replication in DF1 cells.

### 3.6. HSPA8 or HSPA5 Promotes IAV Attachment and Internalization

**Figure 7:**
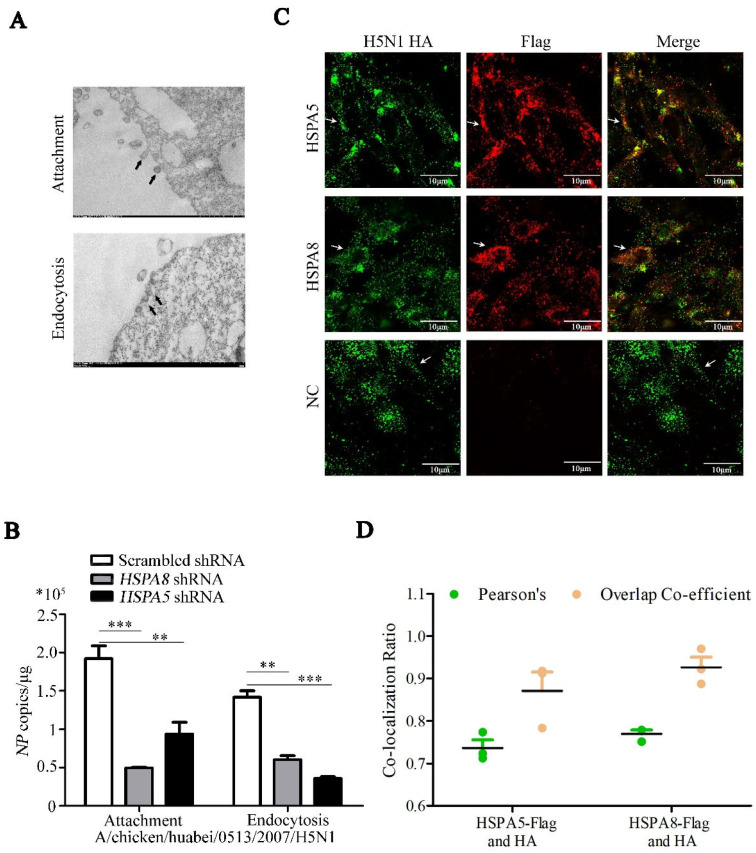
HSPA8 or HSPA5 promotes IAV attachment and internalization. *HSPA5* shRNA-, *HSPA8* shRNA- or scrambled shRNA-transfected DF1 cells were infected with CK/0513 virus (MOI = 1) for 2 h at 4 °C or transferred to 37 °C for 30 min after 2 h. (**A**) Virion attachment and endocytosis were observed by electron microscope. (**B**) *NP* copies were detected by RT-qPCR. *n* = 3, **, *p* < 0.01; ***, *p* < 0.001. (**C**) The colocalization of H5N1 HA and HSPA5 or HSPA8 in DF1 cells was monitored at 48 h after co-transfection without permeabilization. Colocalization (yellow) between HSPA5 or HSPA8 (red) and HA (green) was observed under a Nikon confocal microscope. (**D**) The Pearson’s correlation and overlap coefficient from three independent experiments were analyzed. *n* = 3.

HA binding to the sialic acid-containing cell surface receptor is the first step of IAV initiating the infection process [4]. To further unravel how HSPA8 or HSPA5 affect the early stage of the IAV replication cycle, we compared the attachment and internalization of IAV in the *HSPA5*- or *HSPA8*-specific shRNA-treated DF1 cells and the scrambled shRNA-treated DF1 cells. We first validated Che-Man Chan et al.’s method for detecting virus attachment and endocytosis in wild-type DF1 cells [14]. DF1 cells were infected with CK/0513 H5N1 virus at 4 °C for 2 h; electron microscopy observed that virions attached to cell membranes without internalization (Figure 7A, up). DF1 cells were infected with CK/0513 H5N1 virus at 4 °C for 2 h, followed by incubation at 37 °C for 30 min. After washing away the attached virions with acidic PBS, electron microscopy revealed the internalization of virions (Figure 7A, down). The results show that this method was reliable. Therefore, DF1 cells were transfected with *HSPA5*- or *HSPA8*-specific and scrambled shRNA for 48 h and then infected with CK/0513 H5N1 virus at 4 °C for 2 h. The attached virions of CK/0513 virus were quantified by detecting *NP* copies using a strand-specific RT-qPCR method [14]. This test showed that the IAV virions on the membrane of *HSPA5*- or *HSPA8*-specific shRNA-treated cells was significantly lower than on the scrambled shRNA-treated DF1 cells (Figure 7B). We further performed the virus internalization assay, in which DF1 cells were transfected with *HSPA5*- or *HSPA8*-specific and scrambled shRNA for 48 h. As shown in Figure 7B, the knockdown of *HSPA5* or *HSPA8* in DF1 cells significantly decreased the internalized virions compared to the cells with transfecting scrambled shRNA.

To confirm whether HSPA5 or HSPA8 interact with HA on the cell membrane, the plasmids expressing Flag-tagged *HSPA5* or *HSPA8* (empty NC plasmid) were transfected into DF1 cells, and then infected with CK/0513 H5N1 virus at 4 °C for 2 h. An immunofluorescence assay was performed on DF1 cells without membrane permeability treatment [16]. The absence of membrane permeabilization prevented the intracellular access of specific antibodies and fluorescent dyes, resulting in the observed signal being confined to the plasma membrane surface. Confocal imaging showed the colocalization of HSPA5 or HSPA8 with the HA protein on the cell surface, suggesting that IAV HA might interact with HSPA5 or HSPA8 in attachment process (Figure 7C,D). In addition, the localization of HSPA5 and HSPA8 on the cell surface was also consistent with previous reports [16,19].

In brief, these observations indicate that HSPA5 and HSPA8 interacted with the HA protein to enhance the IAV attachment and internalization.

## 4. Discussion

Influenza A virus (IAV) causes annual epidemics and recurring pandemics, which have a considerable influence on public health and the economy [12,20,21]. Due to the relatively small genome and limited repertoire of encoded proteins, IAV hijacks the host cell machinery to replicate and complete its life cycle [22,23]. Although many studies have been performed to identify host factors necessary for IAV replication via different strategies [10,11,22,24], it is a challenge to identify positive protein interactions [25]. Therefore, it is important to estimate the effects of different approaches and workflows on the sensitivity and accuracy of interaction identifications. In this study, we performed a comparative screening based on immunoprecipitation with mass spectrometry (IP-MS) to evaluate the effects of the expression level of baited protein, as well as virus infection and plasmid transfection, on the identification of protein interactions in human HEK293T, chicken CEF and duck DEF cells (Figure 1A). We found that the plasmid transfection sample had a similar positive interaction ratio and similar biological functions in its IAV HA-interacting host proteins to IAV-infected samples when they had a comparable expression of HA (Figure 2E and Figure 3B). These results suggest the importance of the expression level of baited proteins to the identification of virus–host protein interaction. Our research provides a novel and efficient way to study virus–host protein interactions by IP-MS.

The host range of an IAV is determined by species-specific interactions between the virus and host cell factors [20]. Differences in virus–host protein interactions determine species-specific susceptibility to IAV infection. The IAV HA plays a critical role in virus infection by binding to sialic acid receptors on the cell surface [26]. Typically, a different presentation of sialic acid glycan structure on the host cell membrane is a determinant of the host range of IAV infection [20,27,28]. Here, comparative IP-MS screening allows for the systematic analysis of the differences in IAV HA–host protein interaction networks. In accidental hosts (chicken and human) of IAV, the IAV HA mostly interacts with host proteins involved into cytoskeleton organization and protein folding, but in the reservoir host (duck), the IAV HA-interacting host proteins are enriched in metabolism (Figure 3C). Previous studies indicate that the cytoskeleton facilitates IAV infection by promoting virus entry, vRNPs transport, as well as the correct assembly of new virions [29,30]. These observations could partly explain why rapid IAV infections were detected in humans and chickens, but not in ducks. However, more detailed phylogenetic, structural and functional analyses of IAV HA-interacting host proteins in the three species are needed to verify this hypothesis.

Heat shock protein family A member 5 (HSPA5) and Heat shock protein family A member 8 (HSPA8) belong to the heat shock protein 70 kDa family, which play a key role in maintaining protein folding and in refolding the misfolded and aggregated proteins [31]. Expression profiling reveals the ubiquitous high-level expression of HSPA5 (https://ngdc.cncb.ac.cn/chickengtex/gene/ENSGALG00000001000 (accessed on 29 April 2025)) and HSPA8 (https://ngdc.cncb.ac.cn/chickengtex/gene/ENSGALG00000006512 (accessed on 29 April 2025)) in various avian tissues, indicating their broad functional involvement in chicken physiology. Recent studies revealed the role of HSP70 in various viruses’ modes of replication [31,32,33,34]. For example, HSP70 interacted with IAV PB1/PB2 to enhance RdRP activity, and thereby promoted viral replication or facilitated viral transmission by interacting with the IAV M1 protein [35,36]. We demonstrated that HSPA5 and HSPA8 interacted with IAV HA and facilitated virus attachment and internalization processes during the early stages of the virus’ life cycle in chicken (Figure 7). Moreover, HSPA5 or HSPA8 colocalized with IAV HA in the cell membrane. We therefore hypothesize that HSPA5 and HSPA8 proteins might serve as host cell membrane receptors, being involved in the virus attachment and internalization processes. Our future ability to assess the functions of HSPA5 and HSPA8 in IAV attachment and internalization in vivo will extend knowledge of HA–host interactions in the early stages of IAV.

## 5. Conclusions

Our findings indicate that modulating the expressions of key proteins to levels akin to those in pathological conditions can enhance the rate of positive interactions and yield more authentic interacting factors based on the IP-MS method for screening virus-interacting proteins. Additionally, we identified 582 human, 71 chicken and 71 duck host factors that interact with IAV HA, and found that HA-interacting host factors may play a role in the susceptibility of accidental hosts (human and chicken) to IAV infection, rather than reservoir hosts (duck). We focused on the functions of two heat shock proteins (HSPA5 and HSPA8), elucidating their interactions with IAV HA that facilitate IAV replication by enhancing viral attachment and internalization. These findings extend our knowledge about the mechanisms of IAV entry into host cells and provide target genes for the creation of chickens resistant to avian influenza.

## Data Availability

Data are contained within the article.

## Supplementary Materials

The following supporting information can be downloaded at: https://www.mdpi.com/article/10.3390/pathogens14060535/s1, Table S1. HA-interacting host factors screened in different samples (high, plasmid and virus) of human, chicken and duck species. Table S2. Specific primers used in this study.
